# Vi4-miR-185-5p-Igfbp3 Network Protects the Brain From Neonatal Hypoxic Ischemic Injury via Promoting Neuron Survival and Suppressing the Cell Apoptosis

**DOI:** 10.3389/fcell.2020.529544

**Published:** 2020-11-09

**Authors:** Liu-Lin Xiong, Lu-Lu Xue, Ruo-Lan Du, Hao-Li Zhou, Ya-Xin Tan, Zheng Ma, Yuan Jin, Zi-Bin Zhang, Yang Xu, Qiao Hu, Larisa Bobrovskaya, Xin-Fu Zhou, Jia Liu, Ting-Hua Wang

**Affiliations:** ^1^Institute of Neurological Disease, Translational Neuroscience Center, West China Hospital, Sichuan University, Chengdu, China; ^2^Department of Anesthesiology, The Affiliated Hospital of Zunyi Medical University, Zunyi, China; ^3^School of Pharmacy and Medical Sciences, Division of Health Sciences, University of South Australia, Adelaide, South Australia; ^4^Animal Zoology Department, Institute of Neuroscience, Kunming Medical University, Kunming, China; ^5^Shijiazhuang Maternity and Child Healthcare Hospital, Shijiazhuang, China

**Keywords:** IGFBP3, Vi4, cell apoptosis, hypoxic ischemic encephalopathy, miRNA-185-5p, neuron survival

## Abstract

Neonatal hypoxic ischemic encephalopathy (HIE) due to birth asphyxia is common and causes severe neurological deficits, without any effective therapies currently available. Neuronal death is an important driving factors of neurological disorders after HIE, but the regulatory mechanisms are still uncertain. Long non-coding RNA (lncRNA) or ceRNA network act as a significant regulator in neuroregeneration and neuronal apoptosis, thus owning a great potential as therapeutic targets in HIE. Here, we found a new lncRNA, is the most functional in targeting the Igfbp3 gene in HIE, which enriched in the cell growth and cell apoptosis processes. In addition, luciferase reporter assay showed competitive regulatory binding sites to the target gene Igfbp3 between TCONS00044054 (Vi4) and miR-185-5p. The change in blood miR-185-5p and Igfbp3 expression is further confirmed in patients with brain ischemia. Moreover, Vi4 overexpression and miR-185-5p knock-out promote the neuron survival and neurite growth, and suppress the cell apoptosis, then further improve the motor and cognitive deficits in rats with HIE, while Igfbp3 interfering got the opposite results. Together, Vi4-miR-185-5p-Igfbp3 regulatory network plays an important role in neuron survival and cell apoptosis and further promote the neuro-functional recovery from HIE, therefore is a likely a drug target for HIE therapy.

## Introduction

Hypoxic ischemic encephalopathy (HIE) is an important cause of death and disability in neonates and typically results in serious long-term sequelae including behavioral and cognitive dysfunction, learning difficulties, cerebral palsy and epilepsy (Busl and Greer, 2010; Shankaran et al., 2012; Yang and Kuan, 2015). For the pathological changes, cerebral hypoxia-ischemia (HI) causes the continued injury cascade including cytotoxicity, oxidative stress and mitochondrial disorders, which subsequently lead to neuronal injury and obvious cell death (Brekke et al., 2017). Although the survival rate of HIE has increased as treatments have improved, there is a high risk of permanent neurological deficits in the survivors (Dailey et al., 2013). As a result, HIE affects considerably the health and quality of life of patients and impose significant social and economic burdens. Thus, the key to avoid the HIE-induced long term neurological deficits is to present the neuronal death and enhance synaptic plasticity in the acute time. Hence, this is an unmet medical problem which requires investigation to elucidate mechanisms and to find effective neurotherapeutic targets for HIE. Long non-coding RNAs (lncRNA) are currently thought to be crucial regulators of genomic imprinting, chromatin remodeling, transcription, and cell cycles, and their expression is spatially and temporally restricted to cell types and stages of development (Clark and Blackshaw, 2014). Although their function and mechanism of action are not fully clear, perturbations in lncRNA expression have been implicated in many diseases, including Alzheimer’s disease, heart pathology, and multiple forms of cancer (Clark and Blackshaw, 2014). In the central nervous system (CNS), lncRNAs are particularly abundant and their expression is spatially restricted and temporally regulated (Aprea et al., 2013), playing an important role in brain development. Growing evidence has also demonstrated the functional significance of lncRNAs in neuronal differentiation, maintenance and plasticity (Clark and Blackshaw, 2014). Thus, analysis of lncRNAs may broaden our understanding of the molecular mechanisms of HIE, and provide targets for new therapeutics. It has been documented that there is a crosstalk between lncRNAs and miRNAs (microRNAs), lncRNAs contain miRNA-binding sites, and can function as competing endogenous RNAs (ceRNAs) for miRNAs and protein coding mRNAs, then participate in disease development, such as cardiac hypertrophy and prostate cancer (Chiyomaru et al., 2013; Tay et al., 2014; Wang et al., 2014, 2016). However, the role of this crosstalk in the neurological deficits induced by HIE requires further exploration. Advances in molecular biology technology have led to a more in-depth understanding of HIE pathogenesis.

Therefore, in this study, using gene sequencing and a high throughput functional screening, we have shown that TCONS00044054 (Vi4) plays a crucial role in the neuron survival and cell apoptosis after HIE. Via microRNA sequencing, target scan and RNA22 prediction and quantitative Polymerase Chain Reaction (qPCR) verification, miR-185-5p stands out a site-regulated relationship with Insulin-like growth factor-binding protein 3 (Igfbp3) and Vi4. Moreover, to better understand the potential roles of Vi4, miR-185-5p, and Igfbp-3 crosstalk implicated in the neuron survival, cell apoptosis and further long-term neurological impairments caused by HIE, we further demonstrated their functions via lentivirus-mediated or CRISPER/Cas9 technologies. Taken together, our findings reveal a new pluripotent regulatory circuit that functions via a miRNA competitive mechanism mediated by Vi4.

## Materials and Methods

### Animal Care

The animal protocol of this study has been approved by the Animal Care & Welfare committee of Kunming Medical University. Timed pregnant female Sprague-Dawley rats were purchased from Animal Centre of Kunming Medical University and housed in individual cages. After birth, pups were housed with their dam under a 12 h light/dark cycle, with food and water available *ad libitum* throughout the study. Then 7-days-old SD rat pups (weighing 12–15 g) were used in the later study. MiR-185-5p knock-out (KO) rats were constructed in Cyagen Biosciences (Cyagen, Guangzhou, China). All experiments were performed in consistence with the Guide for the Care and Use of Laboratory Animal published by the United States National Institutes Health.

### Neonatal HI Insult

A modified hypoxic-ischemic model of HIE was generated as previously described (Ferrari et al., 2010). Briefly, 7-days-old (P7) postnatal pups were anesthetized with 3% isoflurane. Following 0.5 cm skin incision in the midline of the neck, the right common carotid artery of each pup was identified, exposed and permanently ligated with an electrocoagulator (Spring Medical Beauty Equipment Co., Ltd., Wuhan, China). After recovering in their dams for 1 h, the pups were then placed in an airtight chamber maintaining hypoxia [8% O_2_, 92% N_2_ at 4 L/min (min)] inside the chamber at a constant 37°C for 2 h. A constant temperature of 37°C was maintained throughout all the procedures. After hypoxia, the animals returned to their dams and the ambient temperature was maintained throughout the entire experimental period. Sham animals underwent anesthesia and the common carotid artery was exposed without ligation and hypoxia.

### Behavioral Studies

Sensorimotor, cognitive, learning, and memory functions were assessed by behavioral tests-NSS test (Shohami et al., 1995), Morris Water maze test (Yang et al., 2017), rotarod (Hamm et al., 1994), Y-maze (Hu et al., 2017), and Open field tests.

### Neurological Severity Score (NSS Score)

Severity of neurological deficit was assessed by use of NSS system (Shohami et al., 1995). The evaluations included the sensory (visual, tactile, proprioceptive), motor (muscle status, abnormal movement), balance tests, and reflex were recorded on a scale of 0–18 (0, normal score; 18, maximal deficit score).

### Morris Water Maze Test

Morris Water maze test was employed to investigate the spatial learning and memory deficits 1 month after HIE as previously described (Yang et al., 2017). In brief, the test was conducted including acquisition phase and probe trial. Initial training was conducted for the first 5 days; during the training period, the rats were guided to locate a hidden and 1 cm submerged platform using peripheral visual information, the water temperature was maintained at 22–24°C. The rats were introduced into the pool for five consecutive days, and four times a day, and 15 to 20 min between training sessions. The rats were given 90 s to locate the platform and were allowed to remain on the platform for 10 s before being removed, while rats that were unable to locate the platform within 90 s were placed on the platform for 10 s before being removed. And the escape latency was recorded. On the 6th day, a probe trial was conducted, in which the platform was removed and the number of crossings over the previous platform location was recorded over one 90 s trial. Tracking System SMART 3.0 (Panlab, Spain) was used to record all the trials and track the movements of the animals automatically.

### Rotarod

Rotarod test was used to assess the animals’ ability to balance, motor coordination and physical condition (Hamm et al., 1994). Each group of rats was subjected to adaptive training for 3 days before the experiment. That means rats were placed on a rotating stick to accommodate the movement on the stick and trained once a day, each time 30–35 revolutions per minute (RPM), training for 5–10 min. After training, rats were placed on a rotating rod suspended 35–40 cm above the table surface, and the speed was 0–40 rpm with the increment of 2 rpm per every 10 s for 3 min. Thereafter, the time of duration (seconds) on the rod was recorded at a constant speed of 40 rpm, the longest time among the three consecutive tests were the final results.

### Y-Maze

In the present study, Y-maze test was performed to detect the distinctiveness, working memory and reference memory of rats. The Y-maze device, provided by Shanghai Xinsoft (Shanghai, China), consisted of three arms including initial, wrong, and food arms. To inspire the animal’s desire to ingest food, all animals should be fasted 1 day in advance. In the adaptation period, the animals were placed in the Y maze, each time for 10 min, adapted to 2–3 times a day, a total of 1 day, and there is no need to put food (this adaption can be synchronized with the fasting period). During 1-day training period, the training frequencies were kept the same for all rats, and the food (chocolate or bait) was placed in one arm of Y maze. Then the door of the other arm was closed, and the rats were placed in the initial arm to find the food. This training lasted for 5–10 min each time. Afterward, the animals could attend the formal test, in this period, the doors of the three arms were opened, the fasted rats were then put into the initial arm, the entry number and duration in each arm within 5 min were recorded with a video camera for each rat, and estimated by SuperMaze V2.0. Under the same moving time on the Y-maze, higher values of the two parameters in the food arm reflected higher spatial memory ability (Hu et al., 2017).

### Open Field Tests

The open field test was selected to further assess locomotion (motor function), autonomous behavior, inquiry behavior and the tension of experimental animals in new environments. The experiment was conducted in a free exploration open-field apparatus, which was 100 cm × 100 cm × 40 cm (length × width × height) and divided into twenty-five 4 × 4 squares. Each rat was carried into the open field and allowed to adapt to the apparatus (10 min daily; three consecutive days) before testing. On the 4th day, rats were re-exposed to the open field for 5 min tests. These rats in stressful situations may lead to a rearrangement of hairs, feathers, and other appendages and sensory stimulation of the skin. In most cases these immediate effects, such as grooming, undoubtedly represent stressful behaviors. Contrarily, rearing (standing upright on the hind legs) is used to indicate a rats’ behavior in a relaxed state. During testing, the time spent grooming and rearing of each rat was recorded by a video camera, and estimated by SuperMaze V2.0.

### Positron Emission Tomography-Computed Tomography (PET-CT)

Positron Emission Tomography-Computed Tomography was carried out to observe the glucose uptake in brains, presented by SUV max. In detail, rats were fasted for 8 h and deprived of water for 4 h before examination. Then rats were anesthetized using Sumianxin II for induction (0.1 mg/kg), and fixed in the supine position in PET/CT scanning table (Discovery 690/Elite, GE, United States). The whole-body CT and PET data were obtained with a standard protocol using AW VolumeShare 5 software for 20 min. The scanning parameters were as follows: voltage for 120 kV, electricity for 260 μA, screw pitch for 0.561, rotational speed for 0.5 s/cycle, thickness and interval for 3.75 mm, 512 × 512 for CT matrix and fov = 50 cm × 50 cm. Thereafter, the PET scan of the same region followed at 2.5 min per bed table position was performed. Attenuation correction and iterative reconstruction of PET images was performed using the CT data and 47 frames of PET cross-sectional images were obtained. Moreover, the CT and PET images were transferred to AW VolumeShare 5 workstation, respectively, and the coronal, sagittal, cross-sectional, and three-dimensional images as well as the fusion images of CT and PET images were obtained. Finally, the PET/CT images were read by two PET/CT reporters via double blind method, and the average value of WB-SUVmax was determined by drawing the ROI of cerebral hemorrhage.

### Tissue Harvest

According to diverse experiments, the preparation of samples was different. After behavioral studies, animals performed for gene analysis were anesthetized, then, their brains were removed, cortex including peri-infarct tissue (approximately 3 mm around the infarcted area of the ipsilateral hemisphere) and hippocampus were separated carefully. The tissues can be stored at −80°C for further use. For morphological detection, the samples were obtained after intracardiac perfusion with 0.9% physiological saline followed by 4% paraformaldehyde (at 4°C, pH 7.4), the brain samples were post-fixed for 5 h at 4°C. The tissue was kept in 30% sucrose in 0.1 Mol (M) phosphate buffer, pH 7.4, for 72 h at 4°C. Then, the brains were embedded in the paraffin. The paraffin-embedded sections were cut into serial horizontal sections (5 μm thickness) and processed simultaneously. For immunocytochemical analysis of neurons, cells were cultured on glass coverslips in 6-well plates. Following washes in PBS, the cells were rinsed and fixed with 4% paraformaldehyde at room temperature for 30 min.

### Triphenyl Tetrazolium Chloride (TTC) Staining and Evaluation of Infarction Volume

The rats were anesthetized with isoflurane and decapitated at 24 h post HI. The brains were rapidly removed and sliced into 2-mm-thick coronal sections in a rat brain matrix (Seino Co., Ltd., Beijing, China). The sections were immediately immersed in 2% 2,3,5-triphenyltetrazolium chlorides (Sigma Co., St. Louis, MO, United States) at 37°C for 30 min in the dark, washed in PBS and then fixed by 4% formaldehyde in phosphate buffered solution. The infarction area of each section was traced and measured using Image J Software (Version 1.43 u; National Institutes of Health, Bethesda, MD, United States). To abolish the error caused by brain edema, we corrected the infarct volume by standard methods as described in the previous report (contralateral hemisphere volume – volume of non-ischemic ipsilateral hemisphere), with infracted volume expressed as a percentage of the contralateral hemisphere. In addition, the brain swelling was determined by subtracting the total volume of the non-ischemic hemisphere from that of the ischemic hemisphere (Hu et al., 2009).

### Hematoxylin and Eosin Staining (HE Staining)

Pathological changes in the brains of rats were observed by HE staining. In brief, the prepared brain sections were exposed to HE staining, and morphology changes in the brain tissues were observed using a light microscope (CX40, Shunyu, Ningbo, China) to detect morphologic changes. Moreover, the average cell size in cortex and hippocampus from five fields of each section (three sections/each animal and five animals/group) was quantitatively analyzed using Image-Pro Plus 6.0 software (Media Cybernetics, Silver Spring, MD, United States). Each section was evaluated by three investigators blinded to the experimental information.

### Nissl Staining

The neuronal cells of the cortex and hippocampus section were visualized by a Nissl staining assay. Briefly, brain slides (5 μm) were stained with 0.1% cresyl violet stain for 1 h at 60°C. The sections were then placed in 0.1% Nissl differentiation, and then they were subsequently washed with distilled water, dehydrated by gradient concentrations of ethanol (70, 80, 90, and 100%), cleared in xylene, and finally coverslipped with neutral balsam. The dark neurons and surviving neurons were observed using a light microscope (magnification, × 200, × 400, Shunyu, Ningbo, China). Five random fields were chosen by a blinded observer and used to quantify positive cell numbers. Cell counts from the left and right hippocampus and cortex on each of the six sections were averaged to provide a single value for each animal.

### Bioinformatics Analysis

The differentially expressed lncRNAs, mRNAs and miRNAs in brain after HI were screened using gene sequencing. Furthermore, in order to predict the relationship among lncRNA, miRNA, and mRNA, ceRNA analysis was performed by Biomarker Technologies in Beijing. In addition, RNA22^1^ was used to predict the binding site between lncRNA and miRNA, the website is. TargetScan^2^ was used to predict miRNAs that target Igfbp3. Venny 2.1^3^ was applied to perform the intersection analysis.

### Luciferase Reporter Assays

Igfbp3 3′UTR luciferase plasmids as well as the corresponding Mut plasmids were generated by RiboBio (Guangzhou, China). The Vi4-ORF-mut was constructed by GeneCopoeia Company (Guangzhou, China). The pmiR-RB-REPORT^TM^ Dual luciferase-expressing vector contained hRlucc DNA encoding *Renilla* luciferase as a reporter and hLucc DNA encoding firefly luciferase as an internal control. Constructs of WT luciferase plasmid contained the full-length 3′-UTR of Igfbp3 mRNA, and the mutant plasmids contained a 3′UTR mutation (mutated from “TCTCTCC” to “AGAGAGG”) could effectively abrogate the binding of Igfbp3 to miR-185-5p. The constructs were confirmed by *Xho*I and *Not*I restriction enzyme digestion and sequencing. Then, 293Tα cells (4 × 10^3^ cells per well) were seeded into triplicate wells of 96-well plates 1 day before transfection. Afterward, the mixture of 3′UTR luciferase plasmids of Igfbp3 or control or port or plasmid (100 ng/ml, Guangzhou RiboBio, China) and miR-185 -5p mimic/mimic-nc (final concentration 80 nM) were transfected into 293Tα cells using SuperFectinTM II *in vitro* DNA Transfection Reagent (Pufei Biotech, China). For the detection between Vi4-ORF-mut and miR-185-5p, Vi4-ORF-mut was transfected into cells 1 day before mimic transfection. 48 h after transfection, luciferase activity was measured with a Dual-Luciferase Reporter Assay Kit (Promega, E1910). The fluorescence value of Renilla fluorescence/firefly is the final relative luciferase activity.

### LncRNA-ORF-Vector Generation

ORF-vector was verified by Enzyme Digest and Electrophoresis and recombinant lentivirus production. Briefly, sequences of lncRNAs were acquired from gene sequencing was sent to GeneChem Company (Shanghai, China) to construct recombinant overexpressed vector (ORF-vector) and then verified by Enzyme Digest and Electrophoresis and sequencing. After that, the lentiviral particles were generated following a standardized protocol using highly purified plasmids and EndoFectin-Lenti^TM^ and TiterBoost^TM^ reagents. Then, the titer of each lentivirus was quantified and lentiviral stocks were stored at −80°C to keep the activity.

### Construction of Igfbp3-sh/Vi4-sh Vectors

Three potential shRNA sequences targeting the Igfbp3 and Vi4 mRNA were designed by GeneChem Company (Shanghai, China), according to the sequence detected in gene sequencing. At the same time, a nonsense shRNA was also designed and synthesized for NC. Then three Igfbp3-sh and Vi4-sh vectors were also provided by GeneChem Company (Shanghai, China). After successful construction, PC12 cells were prepared to single out the most effective shRNA sequence. In brief, when the PC12 cells were 30% confluence, fresh medium containing shRNA lentivirus and 4 μg/ml polybrene, was added to cells. After 3-days transfection, the effects of shRNA were detected by qRT-PCR. Subsequently, the most efficient shRNA-lentivirus-vector was used for the later experiment. In this study, for Igfbp3-shRNA, target sequence (TGACTGATTCCAAGTTCCA) is the most efficient one, for the Vi4-shRNA, target sequence (CGCCAGGTCATCAAGA AGCAA) is the most efficient one, which were used in the later experiment.

### Construction of miR-185 Mimic/Inhibitor

miR-185 mimic/inhibitor [provided by RiboBio (Guangzhou, China)]. The inhibitor, a chemically modified RNA single-strand, is the complementary strand of the miR-185-5p sequence. The sequence of Hsa-miR-185-5p/rno-miR-185-5p is 5′UGGAGAGAAAGGCAGUUCCUGA 3′; the binding sequence of miR-185-5p is 5′TCTCTC3′; miR-185-5p predicts the target sequence to be 5′AGAGA 3′.

### Lentivirus Injection in the Neonatal Rats

To detect the role of Vi4 in the rats with HIE, 3-days old postnatal pups were anesthetized with 3% isoflurane and 5 μl (2 × 10^8^/ml) Vi4-ORF was injected into rats at the right lateral ventricle via microscopic device, and physiological saline was a negative-control (HI-NC). Injection coordinates were 4 mm depth at the following coordinate: 1.5 mm perpendicular to the front cymbal, and further 1.0 mm apart. Infusion was performed at a rate of 100 nl/min. After injection, the glass pipette was left in place for an additional 2 min before being slowly retracted.

### Genotype Identification of miR-185-5p KO Rats

For the rats at 7–10 days after birth, the toes and tail tips were collected and numbered. Then, rats’ genomic DNA was extracted using Transgen’s genomic DNA extraction kit (ee101-12), and PCR detection was performed with the amplification primer: Rat Mir185-F: 5′-CTGATGTGCTCAGGGTGTTGACC-3′; Rat Mir185-R: 5′-GC TGCTGATGTTAGGGAGGAGGC-3′.

### Primary Cortical and Hippocampal Neuron Cultures

The 1-day SD rats were anesthetized and the cortexes/hippocampus were harvested, minced, and isolated by 0.25% trypsin for 10 min at 37°C, then eluted with 10% fetal bovine serum (FBS). Afterward, the tissue suspension was centrifuged at 1000 rpm for 10 min, and complete culture medium (Hyclone) composed of DMEM/HIGH GLUCOSE, 10% fetal calf serum and 1% penicillin-streptomycin solution as used to resuspended the pellets in the bottom. Neurons were then plated in 6-well plates (Corning, United States) coated with poly-d-lysine and laminin (Sigma-Aldrich, St. Louis, MO, United States) at a density of 5 × 10^5^ cells/ml, and incubated at 37°C, 5% CO_2_. In addition, for immunofluorescence staining, we put three cover slips in the 6-well plates before coating. Four hours later, the complete culture medium was replaced with neurobasal medium with the addition of 2% B27 (Invitrogen, Carlsbad, CA, United States). The culture medium was changed the next day, then one-half change was doing every 3 days. The neurons were identified by Tuj1 staining to confirm the purity.

### Transfection of Vi4-ORF or miR-185 Mimic/Inhibitor or Igfbp3-shRNA Into Cortical Neurons

To detect the role of Vi4, miR-185-5p and Igfbp3 on the cell growth or viability, Vi4-ORF-vector, miR-185 mimic/inhibitor [provided by RiboBio (Guangzhou, China)], or Igfbp3-shRNA were transfected into cortical neurons after culturing for 3 days. The transfection system of miR-185-5p was the mixture of 1x riboFECTTMCPBuffber, 100 ng/μlriboFECTTMCP Regent and miR-185-5p mimic (80 nM) or miR-185-5p inhibitor (100 nM), the mixture was added drop-wise to the appropriate wells, respectively. Then, the medium with miRNA transfection was changed immediately before OGD. The transfection system of Vi4-ORF and Igfbp3-sh lentivirus included corresponding lentivirus with no polybrene. Briefly, the lentiviral infection was performed in the titer of two multiplicity of infection (MOI) in primary neurons. After incubation at 37°C for 8 h, the original medium was replaced with fresh medium. eGFP was set as the NC group, and was observed under the Inversed Fluorescent Microscope (Leica, Wetzlar, Germany) to evaluate the transfection efficiency. Moreover, for the rescue experiment between miR-185-5p and Igfbp3, as well as Vi4 and miR-185-5p, we added miR-185-5p mimic into the cells with Vi4-ORF lentivirus or Igfbp3-sh/Vi4-sh into miR-185-5p KO cells. OGD was performed 3 days after transfection. Then the cell growth and viability and cell apoptosis were detected at 24 h post OGD.

### OGD

Neurons were prepared to mimic for HI *in vitro* conditions, according to OGD protocol. Briefly, the cells were washed with 0.01 mM PBS for one time before the medium was changed to glucose-free medium. Then cells were transferred into a hypoxia chamber (Thermo Fisher Scientific, Waltham, United States) with a gas mixture composed of 5% CO_2_ and 95% N_2_ for 2 h. The control cells were incubated normally, and without exposing to OGD.

### Immunofluorescence Staining

Immunocytochemical analysis of Tuj1 was performed to detect the neuron growth. Briefly, for neuronal immunocytochemistry, slices were directly permeated in PBS containing 3% goat serum for 30 min at 37°C. Then, the slices were incubated with primary antibody of Tuj1 (1:200, Rabbit, ABclonal) or Tuj1 (1:200, mouse, ABclonal) and species-specific secondary antibodies of 488 (1:100, goat anti-rabbit, Abbkine) and 594 (1:100, goat anti-mouse, Abbkine). The average length of neuron axon was calculated using Image-Pro Plus 6.0 software (Media Cybernetics, Silver Spring, MD, United States).

### TUNEL Assay

Apoptotic cells were tested by TUNEL assay, the brain tissues and cells were prepared as previously described. In brief, the TUNEL reaction mixture of enzyme solution and labeling solution was added at a ratio of 1:9 (v/v), and the slices of cells were stored at 4°C overnight in the dark. After three washes with PBS, the slices were stained with DAPI for 5 min at room temperature, and images were observed via high-content imaging system (Evons, Thermo, United States). Sixteen fields were randomly selected from each section, and apoptosis was quantified by determining the percentage of TUNEL/DAPI using high-content quantitative system.

### Counting Kit-8 Assays (CCK8)

Neuron viability was assayed by CCK-8 (Dojindo Laboratories, Kumamoto, Japan), according to the manufacturer’s instructions. In brief, neurons were plated at a density of 1 × 10^5^ cells per well in 96-well plates. At the end time, 10 μl CCK-8 solution containing a highly water-soluble tetrazolium salt WST-8 [2-(2-methoxy-4-nitrophenyl)-3-(4-nitrophenyl)-5-(2,4-disulfophenyl)-2H-tetrazolium, monosodium salt] was added into each well, followed by incubation for 3 h at 37°C. Cell proliferation/viability was determined by measuring the OD at 450 nm. Percentage over control was calculated as a measure of cell viability.

### Flow Cytometry

Cell apoptosis was also observed by flow cytometry using a cell apoptosis analysis kit (APC Annexin V Apoptosis Detection Kit with PI). Briefly, at 24 h post OGD, the cells were digested by 0.25% trypsin (EDTA free), followed by centrifuging for 5 min at 1000 rpm. Then, the collected cells were rinsed by 1 × PBS (4°C) for two times, and the cell number was quantified at 1 × 10^6^/sample. Afterward, cells were resuspended by 500 μl 1 × binding buffer, then 5 μl Annexin V-APC and 5 μl PI were mixed with 100 μl cell suspension, followed by incubating for 15 min at room temperature without light. Ultimately, the cells were analyzed by a flow cytometer (Becton Dickinson United States) after being added another 400 μl 1 × Binding Buffer. Apoptosis rate was decided by the sum of the first, second and fourth quadrants.

### Collection of Human Samples

For the serum samples, the patient’s whole blood was gathered through a coagulant tube. After gathering the blood, gently reverse the coagulated tube to mix the blood, then stand them upright at room temperature until the blood was completely coagulated. All serum samples were retrieved and transferred to the laboratory of the Institute of Neuroscience, Kunming Medical University within 2 h after blood collection. After the filtration treatment according to the standard, the serum of 23 people in the BI-3rd group of coagulant tubes was centrifuged for 1000 × 10 min to isolate the serum. Then the collected serum was transferred to 1.5 ml microtubules. All samples were immediately frozen in liquid nitrogen and stored at 80°C. Furthermore, same procedures were performed for the serum obtained from a control group. This experiment was approved by the ethic committee of 2014-2, and all the patients and controls got the informed consent before blood sample collection.

In addition, we also collected a 29-day-old human fetus to culture the primary cortical neurons, this was approved in September 30, 2015 by the Ethics Committee of Kunming Medical University, China (approval No. 2015-9). Informed consent was obtained from the mother. An abort d 29-day-old fetus was collected from the first affiliated hospital of Kunming Medical University and immediately stored on ice. The brain was dissected and placed in 75% alcohol for 2 min. The cortical neurons were then isolated and cultured as described above for the rat neuron culture.

### Quantitative Real-Time Polymerase Chain Reaction (qRT-PCR)

Total RNA was isolated with TRIzol reagent (Takara Bio Inc., Otsu, Japan) and was reverse transcribed to cDNA with the Revert Aid^TM^ First Strand cDNA Synthesis kit (Thermo, United States) and All-in-One miRNA First Strand cDNA Synthesis Kit (GeneCopoeia). The qRT-PCR was then performed to detect the relative expression of mRNA, miR-185-5p and LncRNATCONS00044054 were detected in 24 h after HI and Igfbp3 was detected in 6 h, 24 h and 1 week after HI. The primer sequences were shown as in Table 1. Next, reaction was performed in a DNA thermal cycler (ABI 7300) according to the following standard protocol: one cycle of 95°C for 5 min; 40 cycles of 95°C for 10 s; annealing of 58°C (miR-185-5p) for 20 s, 53°C (Igfbp3) for 10 s, 55°C (lncRNATCONS00044054) for 10 s; and extension of 72°C for 20 s. Relative expressions were calculated with normalization to GAPDH values by using the 2^–ΔΔCt^ method.

**TABLE 1:** The primer information.

	**Forward**	**Reverse**
TCONS00044054 (rat)	5′TGTTCAATT GTCGGTCTTGGTG 3′	5′GAGTTCTGCT GGCTAGTGCTG 3′
Igfbp3 (rat)	5′GGCTCCTT GGGTCGCTTCGT 3′	5′CCCGCCTGAG TTGGACTTCAC 3′
Igfbp3 (human)	5′AGGAAGG AGGAATGGCTTGC 3′	5′CCTCAGTC ATGGCCACAGTT 3′
MiR-185-5p (rat)	HmiRQP0247(GeneCopoeia)	
MiR-185-5p (human)	HmiRQP0247 GAGAGAAAGGCAGTTCCTGAAA	
MiR-380-5p (rat)	HmiRQP0478(GeneCopoeia)	

### Western Blot

Both cortical and hippocampal brain tissues were frozen immediately and stored at 80°C until assessment. To testify the protein expression of Igfbp3 in 6 h after HI, protein was extracted from each group using RIPA lysis buffer (Beyotime, Jiangsu, China) containing 2% of cocktail pill (Roche). Then, the protein concentration was detected by BCA protein assay kit (Beyotime Institute). Afterward, protein (60 μg) was separated by sodium dodecyl sulfate-polyacrylamide gel electrophoresis at 60 V for 30 min and then at 120 V for 1.2 h, and followed by transferring to polyvinylidene fluoride membranes (Millipore, Billerica, MA, United States) over 4 h at 350 mA. After being blocked by 5% non-fat milk for 1 h, the membranes were then incubated with Igfbp3 primary antibody (1:500; Ab6672, rabbit) overnight at 4°C. β-actin (1:2000, A01010, mouse) was set as an internal control. Thereafter, the membranes were rinsed in TBST and incubated with secondary antibody (goat anti-rabbit IgG and goat anti-mouse IgG; ZSGB-BIO, Beijing, China, 1:5000) for 1 h. Finally, after being rinsed in TBST, the membrane was detected using Alpha Innotech (Bio-Rad Laboratories, Berkeley, CA, United States) with ECL.

### Statistical Analysis

All data in the experiment are presented as mean ± SD. Comparisons among groups were analyzed using one-way or two-way repeated-measures ANOVA analysis with the SPSS version 19.0 (IBM Corporation, New York, NY, United States). For multiple group comparison, ANOVA with Tukey’s *post hoc* multiple comparisons were applied. And Student’s *t*-test was used to analyze the data between two groups. *P* < 0.05 was considered statistically significant.

## Results

### The Expression of Vi4 (TCONS00044054) Increased After HIE

**FIGURE 1:**
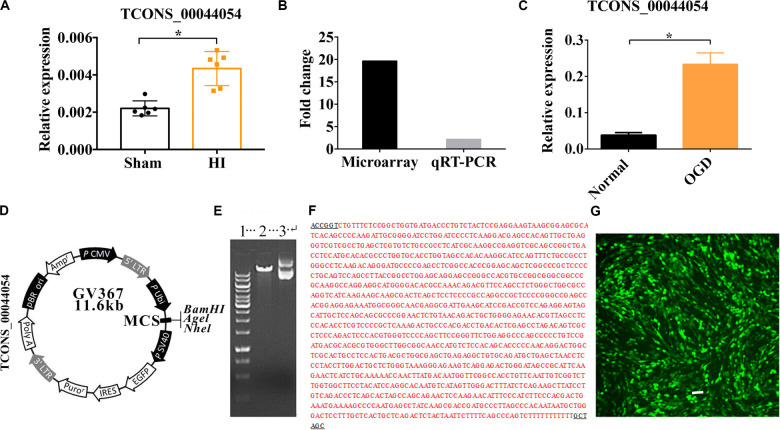
The relative expression of TCONS00044054 (Vi4) in HIE. **(A)** Quantitative histogram for Vi4 expression in sham and HI groups, *n* = 6/group **(B)** The fold change of Vi4 in the microarray and qRT-PCR analysis. **(C)** Quantitative histogram for Vi4 expression in sham and HI groups. **(D,E)** The name of vector was GV367 with *Age*I/*Nhe*I enzymes digestion, showing the plasmid molecular weight of the digested products. 1 was for marker (from up to down: 10 kb, 8 kb, 6 kb, 5 kb, 4 kb, 3.5 kb, 3 kb, 2.5 kb, 2 kb, 1.5 kb, 1 kb, 750 bp, 500 bp, and 250 bp), 2 was for digested vector (11.6 kb), and 3 was for non-digested vector. **(F)** The results of positive clonal sequencing, indicating the clone is ok. **(G)** The transfection of Vi4-ORF into the PC12 cell line, the green color emerged by GFP represents the successful transfection. Scale bar = 100 μm. The relative expression was relative to that of the sham or normal group HI group: hypoxic ischemic group. All data are presented as mean ± SD, *P < 0.05.

The result of qRT-PCR showed that Vi4 was upregulated by HIE (Figure 1A, *P* < 0.05). It is worth noting that Vi4 expression have the same trend as the microarray (Figure 1B). Moreover, it was further found that the expression of Vi4 in OGD was higher than that of normal cortical neurons (Figure 1C, *P* < 0.05). The name of vector was GV367 with *Age*I/*Nhe*I enzymes digestion, showing the plasmid molecular weight of the digested products (Figure 1D). The results of positive clonal sequencing, indicating the plasmid clone is ok (Figures 1E,F). Then, the transfection of Vi4 over-expressed vector (Vi4-ORF) into the PC12 cell line, the green color emerged by GFP represents the successful transfection (Figure 1G).

### Vi4 Overexpression (Vi4-ORF) Protected neurons and Improved the Long-Term Neurological Deficits in Rats With HIE

**FIGURE 2:**
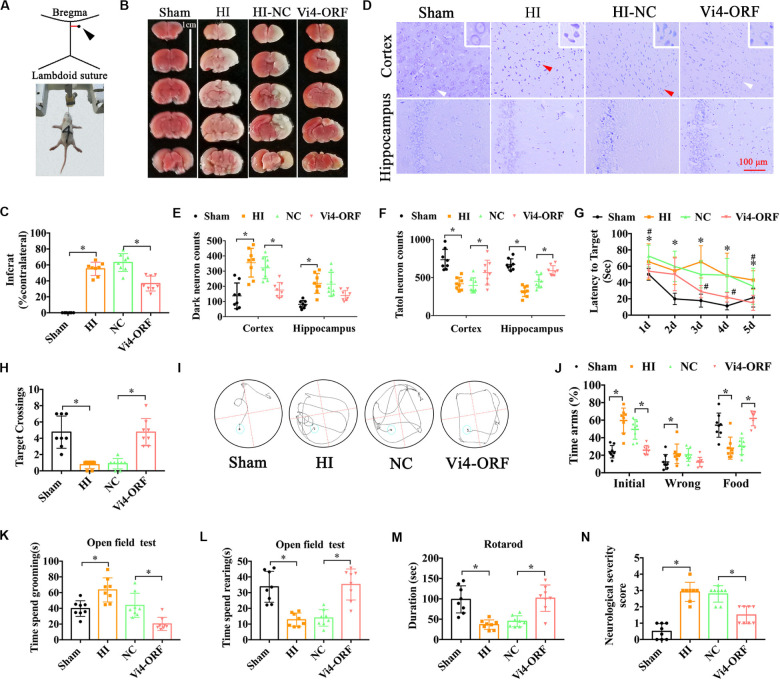
The neurobehavioral tests of rats after intraventricular injection of Vi4-ORF *in vivo*. **(A)** Coordinates of the right ventricle for the Vi4-ORF injection in the P3 rats. **(B)** TTC staining among the sham, HI-NC and Vi4-ORF groups at 24 h after HI. Scale bar = 1 cm. Pale white represents the infarct area, red shows the normal staining. **(C)** Quantitative histogram for the infarct ratio (% contralateral) between NC and Vi4-ORF groups, *n* = 8/group. **(D)** Nissl staining in the groups of sham, HI-NC and Vi4-ORF. The white arrow represents surviving neurons. The red arrow represents dark neurons. Scale bar = 100 μm. **(E,F)** The number of dark neuron and total neuron in cortex and hippocampus among sham, HI, NC and Vi4-ORF groups, *n* = 8/group. **(G)** The latency to target during 5 days training among the sham, HI, NC, and Vi4-ORF groups at 1 month after HI in MWM test. *P < 0.05 vs. Sham. ^#^*P* < 0.05 vs. NC, *n* = 8/group. **(H)** Target crossings in MWM test at 1 month after HI, respectively. **(I)** The walking path of the platform in probe trial of Morris Water Maze test at day 5 of 1 month after HI. **(J)** The time arms (%) of initial, wrong and food arms in the sham, HI, NC and Vi4-ORF groups at 1 month after HI in the Y-maze test, *n* = 8/group. **(K,L)** The time spent grooming and rearing in the open field experiment 1 month after HI, *n* = 8/group. **(M)** The duration of rats on rotary bar in the rotarod test 1 month after HI, *n* = 8/group. **(N)** NSS score among the sham, HI, NC and Vi4-ORF groups 1 month post HI, *n* = 8/group. The N number is the number of biological repeats. HI, hypoxia-ischemia; NC, negative control; H, hours; D, days; MWM, Morris water maze; Vi4-ORF, Vi4 overexpression. All data were presented as mean ± SD, *P < 0.05.

Postnatal day 3 rats were injected with 5 μl (2 × 10^8^/ml) Vi4-ORF or Vi4-negative control (Vi4-NC) into the right lateral ventricle via microscopic device, Vi4-NC was used as a control (HI-NC) (Figure 2A). HIE model was then established 4 days (d) after virus injection. The TTC staining in the HI-NC group showed a clear cerebral infarction on the right side of the brain, and Vi4-ORF significantly decreased the infarction area (Figures 2B,C, *P* < 0.05). In addition, Nissl-staining was used to detect the neuron survival, as a result, Nissl-stained neurons including the normal and dark ones were detected in the both cortex and hippocampus (Figure 2D). Quantitative analysis showed that the number of total neurons was markedly deceased after HI, and the dark neurons was increased as compared with the sham group, while the number of dark neurons in the Vi4-ORF group significantly decreased in comparison to the NC group in those areas (Figure 2E, *P* < 0.05). In addition, Vi4-ORF group showed the more total neurons than NC group both in cortex and hippocampus (Figure 2F, *P* < 0.05). Additionally, spatial learning was evaluated for consecutive 5 days and a probe trial for spatial memory was conducted on the 6th day at two time points of 1-month post HIE. As expected, at both times points the rats injected with Vi4-ORF performed better in learning and memory than that of rats in NC groups (Figures 2G,I, *P* < 0.05). Compared with animals in the HI-NC group, Vi4-ORF-treated rats had significantly more crossings over the previous platform location (Figure 2H, *P* < 0.05). Y-maze was performed to measure the spatial memory of rats 1 month after HIE. As shown in Figure 2J, the time spent in food arm in the Vi4-ORF group was longer than that in the NC group (*P* < 0.05), while time in the initial arm and wrong arm was shorter (*P* < 0.05), indicating that the ventricle injection of Vi4-ORF ameliorated the spatial memory of rats with HIE (Figure 2J, *P* < 0.05). Besides, open field test indicated that Vi4-ORF rats showed less grooming time and significant increase rearing time with respect to the NC group (Figures 2K,L, *P* < 0.05). Similarly, Vi4-ORF-treated rats exhibited increased time on the rotor bar compared to the NC littermates (Figure 2M, *P* < 0.05). However, the treatment with Vi4-ORF of HIE rats had no significant effect on the number of arm entries compared with NC animals. NSS in HI-NC group was obviously increased compared with the sham and Vi4-ORF group at 1 month after HIE, (Figure 2N, *P* < 0.05).

### Vi4 Reduced the OGD-Caused Inhibition of Neuron Growth and Cell Apoptosis

**FIGURE 3:**
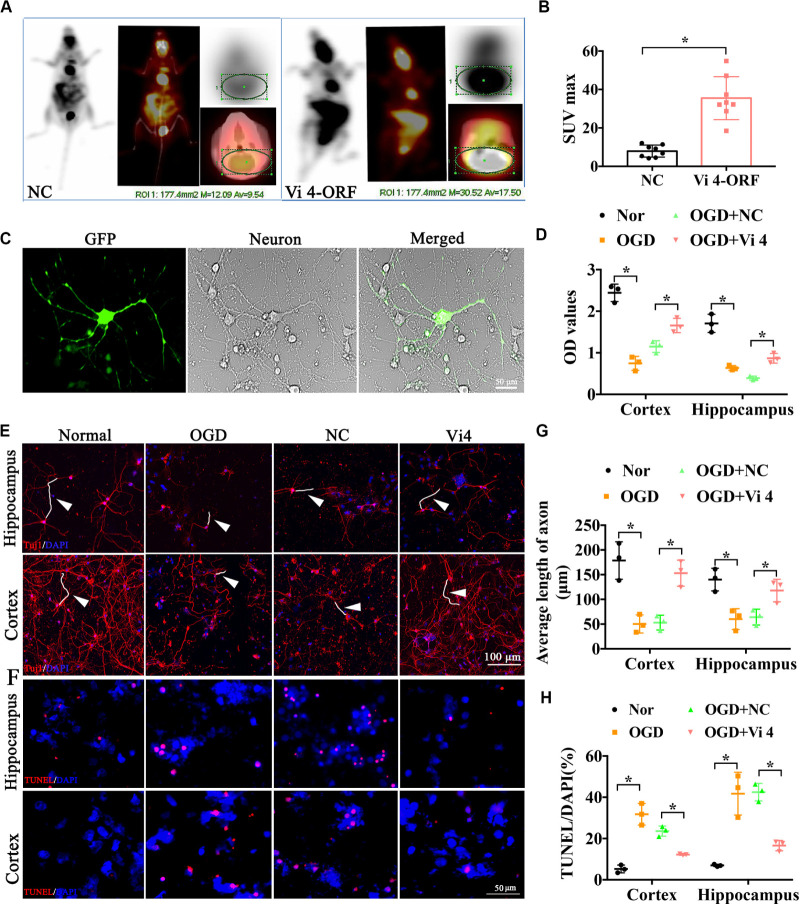
The functional examination on the hippocampal and cortical neurons after the transfection of the Vi4-ORF. **(A)** Images of glucose uptake in the brain by PET-CT in NC and Vi4-ORF groups 2-month after HI. **(B)** The quantified bar chart of SUV max, *n* = 8/group. **(C)** The successful transfection of Vi4-ORF vectors in the cortical neurons, demonstrated by green fluorescence in the cells. Scale bar = 50 μm. **(D)** Cell viability showed by relative CCK8 values in both normal and OGD cells with lentivirus transfection 24 h after OGD in hippocampal and cortical neurons. **(E)** Immunofluorescent staining of Tuj1 to observe the cell state after the ORF-Vi4 vector transfection in the hippocampal and cortical neurons 24 h after OGD. Red fluorescence represents the positive Tuj1 cells, and the blue is the nuclear staining. White arrow represents axon. Scale bar = 100 μm. **(F)** Cell apoptosis detected by TUNEL staining in the cortical neurons 24 h after OGD. Scale bar = 100 μm. Red fluorescence represents TUNEL positive cells, and the blue is the nuclear staining. **(G)** Bar chart for average axon length of cortical and hippocampal neurons at 24 h post OGD. **(H)** Cell apoptosis presented by TUNEL/DAPI (%) in both normal and OGD cells with lentivirus transfection 24 h after OGD. PET-CT, Positron Emission Tomography-Computed Tomography; SUV, Standardized Uptake Value; OGD, oxygen glucose deprivation; Tuj1, neuronal class III β-Tubulin; CCK8, cell counting kit-8; TUNEL, Terminal deoxynucleotidyl Transferase Mediated Nick End Labeling. All data are presented as mean ± SD, *P < 0.05, *n* = 3/group.

Furthermore, PET-CT was carried out to measure the glucose uptake in the brain, quantified by Standardized Uptake Value (SUV) max. As a result, Vi4-ORF injection induced a better glucose uptake in the brain, demonstrated by higher SUV max than that in the NC group (Figures 3A,B, *P* < 0.05). To further explore the effect of Vi4 on the growth of cortical and hippocampal neurons, Vi4-ORF and Vi4-negative control (NC) vectors were transfected into the primary cortical neurons (Figure 3C). The CCK8 results showed that Vi4 increased the cortical and hippocampal neurons viability under OGD in primary neurons compared with NC group (Figure 3D, *P* < 0.05). In addition, through Tuj1 and TUNEL staining, we found that Vi4 reduced apoptotic cells and reserved the length of neuronal axons after OGD in cortical and hippocampal neurons (Figures 3E–H, *P* < 0.05).

### The Upregulation of Igfbp3 by Vi4 in HI Is Competitively Silenced by Negative Regulation of miR-185-5p

**FIGURE 4:**
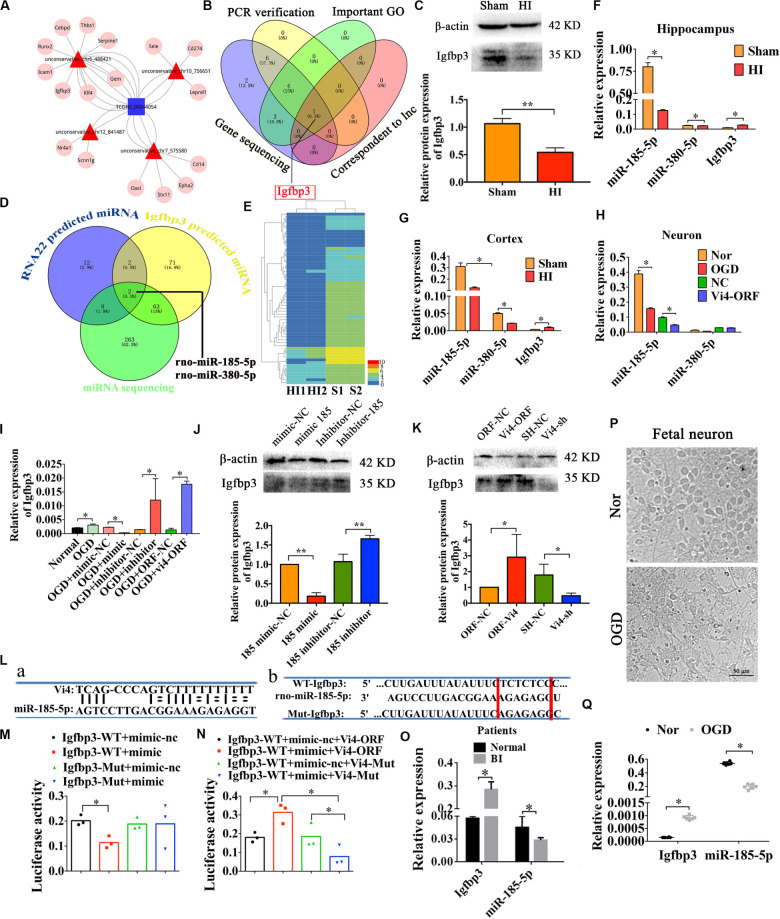
Regulatory relationships among Vi4, miR-185-5p and Igfbp3 in sites and expression. **(A)** ceRNA analysis of Vi4 among diverse miRNAs and mRNAs. The blue square represents Vi4, the red triangles represent miRNAs, and the pink circles represent related genes. **(B)** Intersection of PCR verification, important GO analysis, gene sequencing and correspondent to lnc via bioinformatics analysis for selecting the target gene to Vi4 in the venny 2.1 software. **(C)** Western blot detection of the cortical brain tissues in the sham and HI rats, *n* = 6/group. **(D)** Intersection of RNA22 prediction, TargetScan for Igfbp3-predicted miRNAs and miRNA sequencing to further find the miRNAs that is the most relevant to Igfbp3 and Vi4. **(E)** The heat map of miRNA sequencing between Sham (S1, S2) and HI (H1, H2) groups at 24 h after HI, *n* = 2 in each group. miR-185-5p and miR-380-5p were finally obtained. Red indicates high expression, and blue low expression. **(F)** Relative expression of miR-380-5p, miR-185-5p and Igfbp3 in the sham and HI groups in hippocampus 24 h post HI, *n* = 8/group. **(G)** Relative expression miR-380-5p, miR-185-5p and Igfbp3 in cortex 24 h after HI, *n* = 8/group. **(H)** Relative expression of miR-380-5p and miR-185-5p in neurons 24 h after OGD, *n* = 6/group. **(I)** Relative expression of Igfbp3 in neurons 24 h after OGD, *n* = 6/group. **(J,K)** Western blot detection of the expression of Igfbp3 protein in the cortical neurons in 24 h after OGD, *n* = 6/group. **(L,a)** The binding sites between Vi4 and miR-185-5p predicated by RNA22 software. **(L,b)** The binding sites between miR-185-5p and Igfbp3 3′UTR predicted by TargetScan. The Mut sequences designed for Igfbp3 3′UTR in luciferase reporter assay. **(M,N)** Relative luciferase activities in each group at 48 h after transfection, *n* = 6/group. **(O)** Relative expression of Igfbp3, and miR-185-5p in the serum of BI patients and normal controls. *n* = 23/group. **(P)** The primary cortical neurons from the aborted fetus in the normal and OGD condition. Scale bar = 50 μm. **(Q)** Quantitative detection of the expressional level of miR-185-5p and Igfbp3 in the human neurons after OGD insults, *n* = 6/group. For **(C,F–I,O,Q)**, the relative expression was relative to that of the sham or normal group. For **(J)**, the relative expression was relative to that of the 185-mimic-nc group. For **(K)**, the relative expression was relative to that of the ORF-NC group. WT, wild type; BI, brain ischemia; Mut, mutant. Data are shown as mean ± SD, *P < 0.05.

The analysis of ceRNA was used to explore the relationship among lncRNA, miRNA, and mRNA, to predict the target candidate of Vi4. As predicted by bioinformatics analysis on Vi4 and conjoint analysis of gene sequencing, Igfbp3 was finally determined as the target of Vi4 (Figures 4A,B). This target was further validated by q-PCR verification in OGD cortical neurons after Vi4-ORF transfection, which showed 300-fold upregulation of Igfbp3. In addition, the expression of Igfbp3 was detected in brain of HIE rats at 6 h, 24 h and 1 week after HI (Supplementary Figure 1), and elevated at 24 h after HI. The relative protein expression of Igfbp3 in 6 h after HI group was down-regulation than that of the sham group (Figure 4C, *P* < 0.05). In addition, we asked a question whether Vi4 shares regulatory miRNAs with Igfbp3. Therefore, we applied cross matching of RNA22, TargetScan and miRNA sequencing using rat cortex with HIE to determine common matching miRNAs. As a result, miR-185-5p and miR-380-5p were found most likely miRNAs silencing both Vi4 and Igfbp3 (Figures 4D,E). To confirm the expression of miR-185-5p and miR-380-5p and Igfbp3 after HIE, q-PCR was employed to quantify their levels in the cortex and hippocampus. The results showed that HIE induced significantly increase in the expression of Igfbp3 at 24 h, but decrease in miR-185-5p and miR-380-5p (Figures 4F,G, *P* < 0.05). Then the regulatory relationships among Vi4, miR-185-5p and miR-380-5p and Igfbp3 were also investigated in the primary cortical neurons after OGD. We found that miR-185-5p was markedly down regulated after OGD, and Vi4-ORF treatment can further enhance this effect. However, there was no significant difference in the expression of miR-380-5p, thus miR-185-5p was the focus for the further study (Figure 4H, *P* < 0.05). The relative expression of Igfbp3 in OGD group was up-regulation than that of the normal group (Figure 4I, *P* < 0.05). Moreover, we constructed miR-185-5p mimic and inhibitor to transfect the neurons. We found that miR-185-5p mimic could cause down-regulation of Igfbp3, whereas the miR-185-5p inhibitor or Vi4-ORF could counteract OGD-induced decrease of Igfbp3, indicating both miR-185-5p and Vi4-ORF exhibit competitive regulation of Igfbp3 expression after ODG (Figure 4I, *P* < 0.05). Moreover, the expression of Igfbp3 protein in these groups showed the same trend as qRT-PCR (Figures 4J,K, *P* < 0.05).

Afterward, RNA22 and TargetScan were used to predict the binding sites between Vi4 and miR-185-5p, miR-185-5p and Igfbp3 (Figure 4L). To ascertain whether this observed effect depends on their regulation of the Igfbp3 3′UTR, we constructed luciferase reporters containing Igfbp3 3′UTR [Igfbp3-Wild Type (WT), Igfbp3-Mutant (Mut)]. Luciferase plasmid (Igfbp3-WT, Igfbp3-Mut) and miR-185-5p mimic/miR-185-5p mimic-nc were transfect d into the 293T cell clones. Vi4-ORF-WT/Mut was also constructed. The sequence bound by miR-185-5p is TCTCTC. As a result, in cells transfected with plasmids containing the Igfbp3 3′-UTRs (Figures 4La,b), the relative luciferase activity was significantly decreased after treatment with miR-185-5p, whereas the inhibitory effect of miR-185-5p was abolished in the plasmid containing the mutant 3′-UTRs of Igfbp3 (Figure 4M, *P* < 0.05). For the rescue experiment, Vi4-ORF or Vi4-ORF-Mut was also transfected into the cells with Igfbp3-WT plus miR-185-5p mimic/miR-185-5p-mimic-nc. Vi4 didn’t directly combine with Igfbp3. Vi4 could regulate Igfbp3 by competitively binding miR-185-5p and weaken the combination of Igfbp3 and miR-185-5p. Therefore, overexpression of Vi4 but not the mutant Vi4-ORF-Mut, overcame the decreased luciferase activity of Igfbp3 (Figure 4N, *P* < 0.05). Furthermore, we verified the expressional changes of Igfbp3 and miR-185-5p in the serum of normal and patients with brain ischemia (BI). We found that the expression level of Igfbp3 in BI patients was much higher than that in normal ones, while the expression level of miR-185-5p in BI patients was lower than that of normal subjects (Figure 4O, *P* < 0.05). Additionally, the aborted fetal neurons were further applied to investigate effect of Igfbp3 and miR-185-5p following OGD condition (Figure 4P) and found that the expression of Igfbp3 was obviously elevated in OGD compared with normal neurons (Figure 4Q, *P* < 0.05). Whereas, miR-185-5p had the lowest expression level in OGD (Figure 4Q, *P* < 0.05). These results imply an important role of Vi4 in modulating Igfbp3 by competitively binding miR-185-5p in HI. Therefore, Vi4 is likely to function as a ceRNA for miR-185-5p.

### Neurological Deficits Induced by Neonatal HI Were Improved After miR-185-5p Knockout (KO)

**FIGURE 5:**
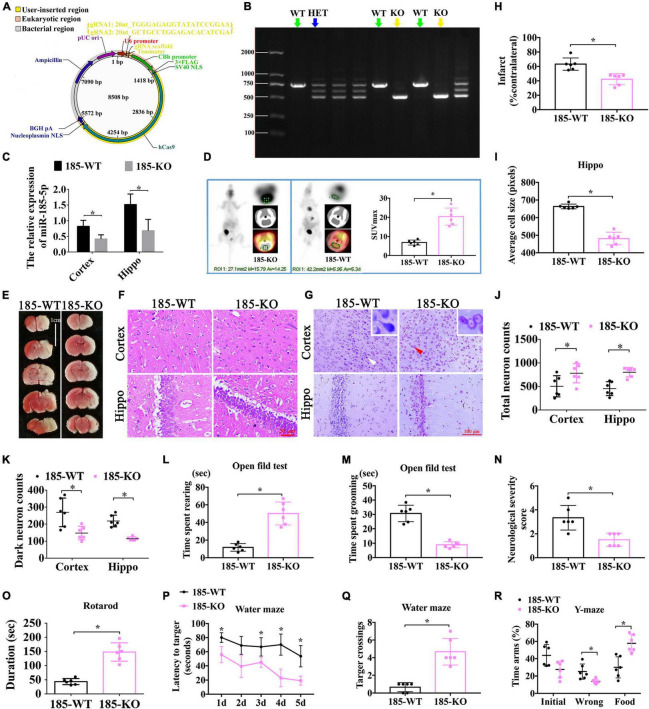
The role of miR-185-5p KO in the improvement of neurological function after HIE. **(A)** The vector map for miR-185-5p vector builder. U6 promoter: Human U6 promoter. {gRNA1: 20nt_TGGGAGAGGTATATCCGGAA; gRNA2: 20nt_GCTGCCTGGAGACACATCGA}: the component entered by user. gRNA scaffold, Chimeric gRNA scaffold; Terminator, U6 terminator; CBh promoter, Chicken beta Act n hybrid promoter; 3xFLAG, 3 tandem flag epitopes; SV40 NLS, SV40 nuclear localization signal; hCas9, Human codon-optimized Cas9; Nucleoplasmin NLS, Nucleoplasmin nuclear localization signal; BGH pA, Bovine growth hormone polyadenylation; Ampicillin, Ampicillin resistance gene; pUCori, pUC origin of replication. **(B)** Electrophoretic band chart for genotype detection. Green arrows represent WT rats, yellow represent KO, and blue arrow represents HET. The markers exhibit 100 bp, 250 bp, 500 bp, 750 bp, 1000 bp, and 2000 bp, respectively. **(C)** The relative expression of Igfbp3 in the rats of 185-WT and 185-KO. The relative expression was relative to that of the 185-WT in cortex group. **(D)** The glucose-uptake images in the brain and SUV max detected by PET-CT 2-month post HIE. **(E,H)** TTC staining and quantitative analysis of infarct ratio, respectively, in the rats of 185-WT and 185-KO. Scale bar = 1 cm. Pale color represents infarct area, *n* = 6/group. **(F,I)** HE staining and the cell size analysis of cortex and hippocampus, respectively, in the rats of 185-WT and 185-KO. Scale bar = 50 μm, *n* = 6/group. **(G)** Nissl staining in cortex and hippocampus between 185-WT and 185-KO groups. The white arrow represents surviving neurons. The red arrow represents dark neurons. Scale bar = 100 μm. **(J,K)** Quantitative histogram for total neuron and dark neuron in cortex and hippo in these groups, *n* = 6/group. **(L,M)** The time spent rearing and grooming in the open field test 1 month after HIE, respectively, *n* = 6/group. **(N)** NSS score at 1 month after HIE. **(O)** The duration of on the rotarod bar 1 month after HIE, *n* = 6/group. **(P)** The latency to target for the first 5 days training in the MWM test, *n* = 6/group. **(Q)** Target crossings in MWM test in the 6th day of testing. **(R)** The time spent in the initial, wrong and food arms of Y-maze at 1-month post HIE, *n* = 6/group. HET: heterozygote. 185-WT: miR-185-5p wild type. 185-KO: miR-185-5p knockout. PET-CT, Positron Emission Tomography-Computed Tomography; SUV, standardized uptake value; HE, hematoxylin-eosin; Hippo, hippocampus. All data are presented as mean ± SD, *P < 0.05.

To investigate the function of miR-185-5p in rats with HIE, we established the miR-185-5p KO rats through CRISPR/CAS9 technology. Briefly, two single gRNA (sgRNA) action targets for miR-185 gene were designed. Oligonucleotide chains were synthesized according to the sticky end formed by sgRNA vector through *Bsa*I, then the chains were connected to pRP [CRISPR]-hCas9-U6 carrier after annealing. Construction of sgRNA vector was completed and was confirmed by sequencing (Figure 5A). After microinjection for F0, we began to reproduce to acquire Fragment 1 (F1), and verified the genetic modification of miR-185-5p caused by CRISPR/CAS9 via PCR gel electrophoresis to determine WT, homozygote (KO), and heterozygote (HET) off springs. WT and KO were used for the later experiment (Figure 5B). The result of qRT-PCR showed that there was lower expression of miR185-5p in the cortex and hippocampus of the miR-185-5p-KO rats than that of WT rats (Figure 5C, *P* < 0.05). Intracerebral glucose intake was observed by PET-CT, and quantified by the SUV-max. The glucose intake was increased in miR-185-5p-KO, compared to that of WT rats (Figure 5D, *P* < 0.05). As shown in Figure 5E, the TTC staining in the miR185-5p-WT group showed a clear cerebral infarction on right side of the brain, and the cerebral infarction as significantly reduced in the miR-185-5p-KO group, as demonstrated by the reduced infarct ratio (% contralateral) (Figure 5H, *P* < 0.05). Because of the swelling of cells after HIE injury and the cell layer of the hippocampus was thickened. However, the swelling of cells was attenuated in miR-185-5p-KO group. HE staining of the hippocampus showed that the cell layer size of the miR-185-5p-KO group was significantly thinner than that of the miR185-5p-WT group (Figures 5F,I, *P* < 0.05). Besides, Nissl-stained neurons were observed in cortex and hippocampus from miR185-5p-WT and miR185-5p-KO rats (Figure 5G). The miR185-5p-KO group showed the more total neurons and the lower dark neurons in both cortex and hippocampus than that of miR185-5p-WT group (Figures 5J,K, *P* < 0.05).

In order to observe the exploratory behavior, general activity and anxiety of experimental animals in new environments, the open field experiment was performed. Compared with WT rats, the rearing time spent by KO rats was clearly increased (Figure 5L, *P* < 0.05), but the grooming time spent by KO rats was largely decreased (Figure 5M, *P* < 0.05). In addition, compared with animals in the WT group, miR-185-5p KO rats had a reduced NSS score at both 1 month after HIE (Figure 5N, *P* < 0.05). Additionally, the running time on the rotarod by miR-185-5p KO rats was longer than that by WT rats (Figure 5O, *P* < 0.05) in the rotarod test, indicating these KO rats possessed better motor function and coordination ability 1 month after HIE. Similarly, spatial learning and memory were evaluated via Morris water maze at 1-month post HI induction. As a result, the KO rats performed better than the WT rats in finding the platform with a better learning performance through 5 days of training (Figure 5P, *P* < 0.05). On the 6th day, these KO rats also exhibited more crossings to the previous platform, indicating a better spatial memory (Figure 5Q, *P* < 0.05). In the test of Y-maze, KO animals spent shorter time in the wrong arm and longer time in the food arm than the WT animals. However, there was no significant difference in each arm entries (Figure 5R, *P* > 0.05). All these findings above suggest that knockout of miR-185-5p could ameliorate the motor, learning and memory dysfunctions induced by HIE.

### miR-185-5p KO Decreased Cell Apoptosis and Enhanced Cell Viability in Primary Cortical Neurons and Hippocampal Neurons After OGD Associated With Vi4 and Igfbp3 Regulation

**FIGURE 6:**
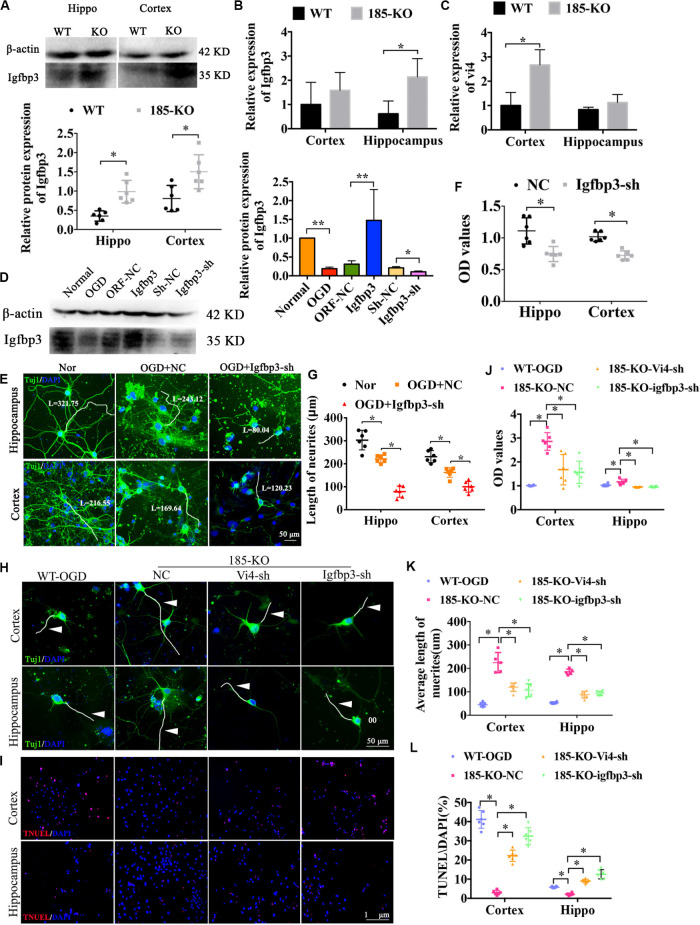
The role of miRNA-185-5p KO in the growth of neurons and cell apoptosis as well as the regulatory relationship with Vi4 and Igfbp3 in function. **(A)** Western blot detection of the expression of Igfbp3 protein in the cortical and hippocampal brain tissues of the WT and 185-KO rats, *n* = 6/group. The relative expression was relative to that of the 185-WT in hippocampus group. **(B,C)** The expression of Igfbp3 and Vi4 in cortex and hippocampus from WT and 185-KO rats. The relative expression was relative to that of the 185-WT in cortex group. **(D)** Western blot detection of the expression of Igfbp3 protein in the cortical neurons in the groups of normal, OGD, NC, Igfbp3 and Igfbp3-sh. The relative expression was relative to that of the sham or normal group. **(E,G)** Immunofluorescent staining of Tuj1 in Nor, OGD + NC, and OGD + Igfbp3-sh groups to detect the cell phenotype and axon growth. The neurons are stained by green colors, which are Tuj1 positive. Blue represents the nucleus. Scale bar = 50 μm. **(F)** Relative CCK8 values in the cortical and hippocampal neurons between NC and Igfbp3-si groups, *n* = 6/group. **(H)** Immunofluorescent staining of Tuj1 in the cortical and hippocampal neurons, respectively, in the groups of WT, miR-185-5p-KO + NC (185-KO-NC), miR-185-5p-KO + Vi4-sh (185-KO-Vi4-sh) and miRNA-185-5p-KO + Igfbp3-sh (185-KO-Igfbp3-sh) 24 h after OGD. Scale bar = 50 μm, green staining represents the Tuj1 positive cells, and the nucleus s stain d by blue. White arrow represents axon. **(I)** TUNEL staining in the WT, miR-185-5p-KO + NC (185-KO-NC), miR -185- 5p-KO + Vi4-sh (185-KO-Vi4-sh) and miRNA-185-5p-KO + Igfbp3-sh (185-KO-Igfbp3-sh) groups 24 h after OGD in both cortical and hippocampal neurons. Scale bar = 100 μm. Apoptotic cells are stained by red color, and the nucleus is stained by blue. **(J–L)** Quantitative histograms of relative CCK8 values, the average length of axon and cell apoptosis presented by TUNEL/DAPI (%). Nor, normal; Vi4-sh, Vi4-shRNA, interference of Vi4 by shRNA; Igfbp3-sh, interference of Igfbp3 by shRNA. All data are shown as mean ± SD, ^∗^*P* < 0.05, ^∗∗^*P* < 0.01, *n* = 6/group.

As shown in Figure 6A, after knocking out miR-185-5p in the rats, the protein level of Igfbp3 was significantly upregulated when compared to the WT rats, as indicated by western blot detection in both cortex and hippocampus (Figure 6A, *P* < 0.05). The expression of Igfbp3 mRNA in hippocampus of miR-185-5p-KO rats was obviously increased compared to WT rats (Figure 6B, *P* < 0.05). Similarly, the mRNA level of Vi4 was significantly upregulated in cortex of miR-185-5p-KO rats when compared to the WT rats (Figure 6C, *P* < 0.05). Consequently, Igfbp3 siRNA was constructed to silence the expression of Igfbp3. The result of WB showed the expression of Igfbp3 protein was remarkably reduced after interfering Igfbp3 (Figure 6D, *P* < 0.05). After OGD, the cortical and hippocampal neurons were damaged significantly compared with the normal neurons, especially the neurites, as indicated by Tuj1 staining. Igfbp3 knockdown aggravated the neuron injury, as demonstrated by shorter neurites and lower cell viability detected by CCK8 assay as compared with the NC group (Figures 6E–G, *P* < 0.05). These demonstrated that Igfbp3 played an important role in neuroprotection after OGD. Furthermore, to investigate whether miR-185-5p regulates cell growth through modulating Igfbp3 or functioning together with Vi4, we performed immunofluorescent staining of Tuj1 and TUNEL as well as CCK8 in WT and KO neurons from both cortex and hippocampus with OGD. For the rescue experiment, interference of Vi4 by shRNA (Vi4-shRNA) or Igfbp3-shRNA was added to KO cells. As a result, OGD induced serious neuron damage, such as cell apoptosis and axonal injury, while miR-185-5p KO reduced cell apoptosis and showed better axon growth. This protective role was abolished by the inhibition of Vi4 or Igfbp3 (Figures 6H,I), as demonstrated by lower CCK8 values and shorter neurites as well as more cell apoptosis in both cortical and hippocampal neurons (Figures 6J–L, *P* < 0.05). These findings further ascertain that miR-185-5p KO could protect neurons from OGD via modulating Igfbp3 and Vi4.

### Inhibition of Vi4 and Igfbp3 Aggravated the Neuron Damage and Neurological Deficits in Rats With HIE

**FIGURE 7:**
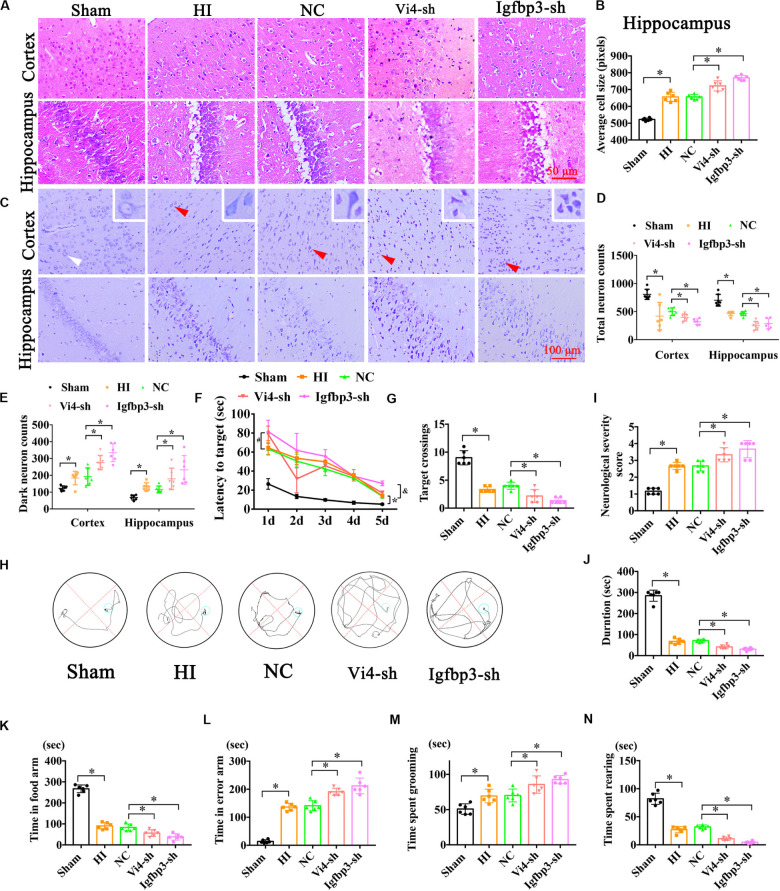
The neurobehavioral tests of rats after intraventricular injection of Vi4-sh and Igfbp3-sh *in vivo*. **(A,B)** HE staining and the cell size analysis of cortex and hippocampus, respectively, in the rats of Sham, HI, NC, Vi4-sh and Igfbp3-sh. Scale bar = 50 μm, *n* = 6/group. **(C)** Nissl staining in cortex and hippocampus among sham, HI, NC, Vi4-sh and Igfbp3-sh groups. The white arrow represents surviving neurons. The red arrow represents dark neurons. Scale bar = 100 μm. **(D,E)** Quantitative histogram for total neuron and dark neuron in these groups, *n* = 6/group. **(F)** The latency to target during 5 days training among the sham, HI, NC, Vi4-sh and Igfbp3-sh groups after HI in MWM test. *P < 0.05: HI vs. Sham.^ #^*P* < 0.05: Vi4-sh vs. NC. ^&^*P* < 0.05: Igfbp3-sh vs. NC, *n* = 6/group. **(G)** Target crossings in MWM test after HI, respectively, *n* = 6/group. **(H)** The walking path of the platform in probe trial of Morris Water Maze test at day 5 after HI. **(I)** NSS score among the sham, HI, NC, Vi4-sh and Igfbp3-sh groups post HI, *n* = 6/group. **(J)** The duration of rats on rotary bar in the rotarod test after HI, *n* = 6/group. **(K,L)** The time arms of food and error arms in the sham, HI, NC, Vi4-sh and Igfbp3-sh groups after HI in the Y-maze test, *n* = 6/group. **(M,N)** The time spent grooming and rearing in the open field experiment after HI, *n* = 6/group. All data were presented as mean ± SD, ^∗^*P* < 0.05.

Postnatal day 3 rats were injected with 5 μl (2 × 10^8^/ml) Igfbp3-sh or Vi4-sh or sh-NC into the right lateral ventricle via microscopic device. The results of HE staining showed that the cell layer size of the Igfbp3-sh group was significantly thicker than that of the NC group in the cortex and hippocampus (Figures 7A,B, *P* < 0.05). Moreover, Nissl-staining revealed (Figure 7C) that the number of total neurons was obviously reduced in HI group, and the dark neurons was increased in comparison to sham group. Whereas, the number of total neurons was remarkably decreased in the Igfpb3-sh group as compared with NC group (Figure 7D, *P* < 0.05). Furthermore, Igfpb3-sh group showed more dark neurons in both cortex and hippocampus than that of NC group (Figure 7E, *P* < 0.05). In addition, spatial learning was evaluated for consecutive 5 days and a probe trial for spatial memory was conducted on the 6th day at post HIE. As expected, at both times points the rats injected with Igfbp3-sh performed worse in learning and memory than rats in the sham or NC groups (Figures 7F,H, *P* < 0.05) Compared with the NC group, the rats of Igfbp3-sh group had significantly less crossings over the previous platform location (Figure 7G, *P* < 0.05). Additionally, NSS in Igfbp3-sh group was obviously increased compared with the NC group after HIE (Figure 7I, *P* < 0.05). Similarly, Igfbp3-sh rats exhibited decreased time on the rotor bar compared to the NC littermates (Figure 7J, *P* < 0.05). Furthermore, the Y-maze was performed to measure the spatial memory of rats 1 month after HIE. The time spent in food arm in the Igfbp3-sh group was shorter than that in the NC group (*P* < 0.05), while time in the error arm was longer (*P* < 0.05), indicating that the ventricle injection of Igfbp3-sh damaged the spatial memory of rats with HIE (Figures 7K,L, *P* < 0.05). Open field test indicated that Igfbp3-sh rats showed more grooming time and less rearing time compared to the NC group (Figures 7M,N, *P* < 0.05). Moreover, knocking down Vi4 showed the same trend as Igfbp3-sh, which increased neuronal apoptosis and worsen the motor and cognitive deficits in rats with HIE (Figure 7, *P* < 0.5).

## Discussion

Accumulating evidence showed that lncRNAs and miRNAs played important roles in physiological and pathological conditions. As a result, new drugs were under development to target miRNAs and lncRNAs for the treatment of cancers and other diseases. In this study, we reported that a novel lncRNA (Vi4) as upregulated in the brain of HIE rats and in cortical neurons under OGD conditions, promoted neurite growth and reduces cell apoptosis of cortical and hippocampal neurons after HI. We have elucidated a functional mechanism of Vi4 which upregulated the Igfbp3 gene via competitively binding miR-185-5p in HI. *In vivo* studies showed that, overexpression of Vi4 or silencing (or KO) of miR-185-5p improved long-term motor functions and reduced the deficiencies in learning and memory of HIE rats.

In the present study, we have successfully established a HIE model in neonatal rats and found Vi4 has a function of promoting the cell growth and neuroprotection in rats with HIE. Accumulating evidence has indicated that the brain was the organ with the most abundant and varied lncRNAs which also existed in the other parts of mammalian body except reproductive cells (Goff et al., 2015). Many lncRNAs were specifically expressed in nerve cells with different levels of expression in different brain regions (Aprea et al., 2013; Luo et al., 2013). For example, a high level of expression of lncRNA HOTAIR facilitated the onset of ischemic infarct induced by hypoxia (Yang and Lu, 2016). MALAT 1 protects brain microvasculature and parenchyma after brain ischemic injury by suppressing endothelial cell death and inflammation (Zhang et al., 2017). The present study has for the first time identified the novel function of Vi4 involved in neuroprotection in the rat model of HIE. It is highly expressed in the brain of HI and in neurons with OGD. Lentivirus-mediated overexpression of Vi4 promoted growth of neurites, increased viability and reduced apoptosis of primary neurons of cortex and hippocampus after OGD *in vitro*. Consistently, neonatal HIE rats with Vi4-ORF treatment showed a reduced infarction size and better performance in motor behavior and cognitive functions after acute and long-term HI. PET-CT showed these rats treated with Vi4-ORF also exhibited more glucose-uptake in the brain. Thus, our results indicated that a higher level of Vi4 was associated with the recovery of neurological deficits induced by neonatal HIE. Through conjoint analysis of bioinformatics prediction and PCR verification, we found that Igfbp3 was closely correlated with Vi4 in both regulatory sites and function. We observed that the overexpression of Vi4 was enough to increase the mRNA level of Igfbp3 and promote neurological recovery. It is known that Igfbp3 on the surface of vascular endothelial cells may help attracting IGF-I, which stimulates angiogenesis (Danis and Bingaman, 1997), and the level of Igfbp3 mRNA in cerebral vascular endothelial cells is increased at 1 h after HI, and reaches its highest level at 24 h after recovery (Lee et al., 1999). In contrast, after HI (1, 5, 24, and 72 h), neuronal Igfbp3 mRNA expression levels in the area of normal middle cerebral artery supply decreased significantly within 24 h (Lee et al., 1999), which was consistent with our findings of down-regulation of Igfbp3 protein expression at 6 h after HI. These results suggested that Igfbp3 downregulation was detrimental to neurological function after HIE injury. Accumulating evidence showed that Igfbp3 could promote axonal growth, cellular proliferation and differentiation (Devesa et al., 2014 and Kartal et al., 2016). In this sense, the lack or deficiency of hormones may lead to acute and subacute consequences, such as hypoglycemia, growth retardation, mental-motor deficiency, disturbances in cognitive functions and cardiovascular system (Volpe, 2001), indicating an important role of Igfbp3 in HIE. Our data in the present study showed that Vi4 was an upstreaming positive regulator of the Igfbp3 gene in HIE was consistent with these published results, indicating the Vi4-Igfbp3 signal was a critical neuroprotective factor in HI injury. Furthermore, to answer a question of how Vi4 functions with Igfbp3, and if some other pathways exist in the regulation between Vi4 and Igfbp3, we performed double prediction via RNA22 and TargetScan, then combined with luciferase reporter assay and functional detection. Our results indicated that Vi4 shared the response element of Igfbp3 with miR-185-5p. To confirm our prediction, we constructed miR-185-5p KO rats, and examined their regulatory relationship *in vivo* in the model of HIE and *in vitro* neurons under OGD. We have found that rats with miR-185-5p KO perform better in the long-term spatial learning and memory and motor tests. In addition, after miR-185-5p knockout, the cortical and hippocampal neurons grew better than WT neurons, and cell apoptosis was also decreased after OGD. Furthermore, these positive effects were partially reversed by interfering with Vi4 or Igfbp3. Evidence in the past few years has shown that miR-185 was a novel tumor suppressor and was one of the most well-defined miRNAs in cancer biology. It was associated with cell proliferation and apoptosis and plays a major role in tumorigenesis and tumor progression (Dong-Xu et al., 2015; Sun et al., 2017). However, there is no study to show it is associated with HIE. Our result in present study indicated that miR-185-5p could be a therapeutic target in HIE, and Vi4 may function as a ceRNA by competitively binding miR-185-5p, and modulate Igfbp3 in the process of HI-induced neuronal injury. In order to further validate this finding, we also verified their expressional levels in patients with brain ischemia, and indeed showed that the level of Igfbp3 was increased and miR-185-5p decreased after brain ischemia in the blood of patients. The changes of Igfbp3 and miR-185-5p after brain ischemia may represent early compensatory mechanisms for self-protection in the body (Purushothuman and Stone, 2015).

Taken together, our research has demonstrated that Vi4 and miR-185-5p act as key regulators of the Igfbp3 gene, whereby Vi4 positively promotes its gene expression but miR-185-5p negatively counteracts Vi4. Our study has also elucidated important roles of Vi4-miR-185-5P-Igfbp3 signaling network in regulating neuron survival and cell apoptosis after HIE, showing Vi4-Igfbp3 signal promotes functional recovery whereas miR185-5p aggravates the brain damage after HIE. Vi4-miR185-5p-Igfbp3 could be potential therapeutically targets for HIE treatment.

## Data Availability Statement

The datasets generated for this study can be found in the Vi4-miR-185-5p-lgfbp3 network protects the brain from neonatal hypoxic ischemic injury via promoting neuron survival and suppressing the cell apoptosis.

## Ethics Statement

The studies involving human participants were reviewed and approved by the Ethics Committee of Kunming Medical University, China (Approval No. 2015-9) in September 30, 2015. The patients/participants provided their written informed consent to participate in this study. The animal study was reviewed and approved by the Animal Care and Welfare Committee of Kunming Medical University. Written informed consent was obtained from the owners for the participation of their animals in this study and individual(s) for the publication of any potentially identifiable images or data included in this article.

## Author Contributions

L-LXi, L-LXu, R-LD, H-LZ, Y-XT, ZM, YJ, Z-BZ, YX, QH, LB, X-FZ, JL, and T-HW performed the material preparation and data collection and analysis. L-LXi, L-LXu, H-LZ, QH, and T-HW wrote and revised the first draft of the manuscript. All authors contributed to the study conception and design, commented on previous versions of the manuscript, and read and approved the final manuscript.

## Conflict of Interest

The authors declare that the research was conducted in the absence of any commercial or financial relationships that could be construed as a potential conflict of interest.

## Footnotes

1
https://cm.jefferson.edu/rna22/Interactive/

2
http://www.targetscan.org/vert_72/

3
http://bioinfogp.cnb.csic.es/tools/venny/
