# Corrigendum: Vi4-miR-185-5p-Igfbp3 Network Protects the Brain From Neonatal Hypoxic Ischemic Injury via Promoting Neuron Survival and Suppressing the Cell Apoptosis

**DOI:** 10.3389/fcell.2022.852539

**Published:** 2022-02-16

**Authors:** Liu-Lin Xiong, Lu-Lu Xue, Ruo-Lan Du, Hao-Li Zhou, Ya-Xin Tan, Zheng Ma, Yuan Jin, Zi-Bin Zhang, Yang Xu, Qiao Hu, Larisa Bobrovskaya, Xin-Fu Zhou, Jia Liu, Ting-Hua Wang

**Affiliations:** ^1^ Institute of Neurological Disease, Translational Neuroscience Center, West China Hospital, Sichuan University, Chengdu, China; ^2^ Department of Anesthesiology, The Affiliated Hospital of Zunyi Medical University, Zunyi, China; ^3^ School of Pharmacy and Medical Sciences, Division of Health Sciences, University of South Australia, Adelaide, SA, Australia; ^4^ Animal Zoology Department, Institute of Neuroscience, Kunming Medical University, Kunming, China; ^5^ Shijiazhuang Maternity and Child Healthcare Hospital, Shijiazhuang, China

**Keywords:** hypoxic ischemic encephalopathy, Vi4, miRNA-185-5p, IGFBP3, neuron survival, cell apoptosis

In the original article, there was a mistake in Figure 5A as published. In the original Figure 5A, there was only sequence of one gRNA, but the sequences of two gRNAs should be provided in the vector map for knocking out miR-185-5p, thus we have updated the sequences of two gRNAs in the corrected Figure 5A. The corrected Figure 5A appears below.

**FIGURE 5:**
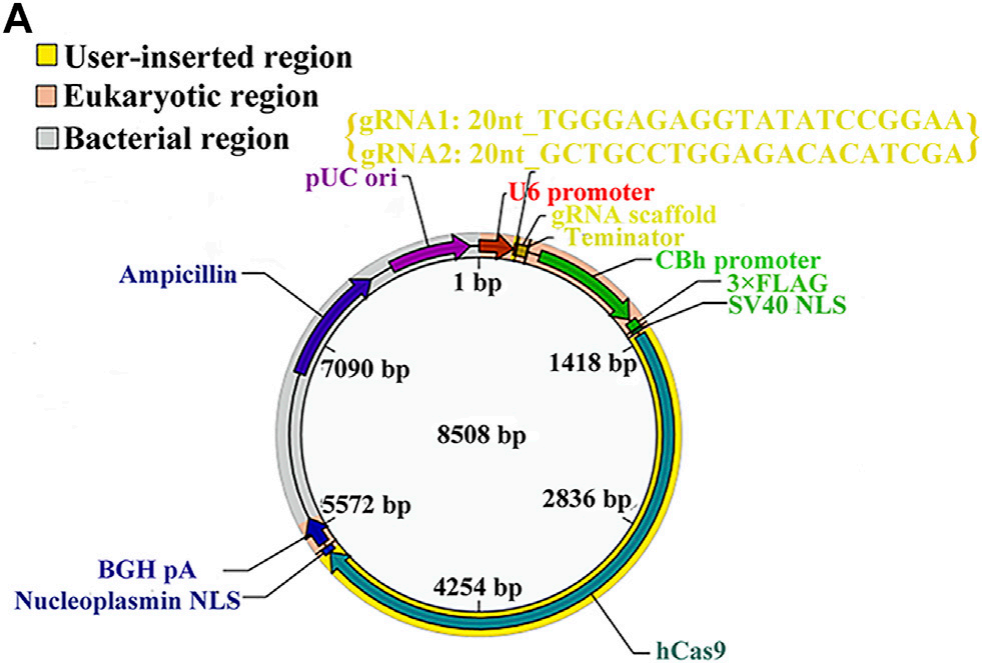
**(A)** The vector map for miR-185-5p vector builder. U6 promoter: Human U6 promoter. {gRNA1: 20nt_TGGGAGAGGTATATCCGGAA; gRNA2: 20nt_GCTGCCTGGAGACACATCGA}: the component entered by user. gRNA scaffold, Chimeric gRNA scaffold; Terminator, U6 terminator; CBh promoter, chicken beta act n hybrid promoter; 3xFLAG, three tandem flag epitopes; SV40 NLS, SV40 nuclear localization signal; hCas9, human codon-optimized Cas9; Nucleoplasmin NLS, nucleoplasmin nuclear localization signal; BGH pA, bovine growth hormone polyadenylation; Ampicillin, ampicillin resistance gene; pUCori, pUC origin of replication. **(B)** Electrophoretic band chart for genotype detection. Green arrows represent WT rats, yellow represent KO, and blue arrow represents HET. The markers exhibit 100, 250, 500, 750, 1,000, and 2,000 bp, respectively. **(C)** The relative expression of Igfbp3 in the rats of 185-WT and 185-KO. The relative expression was relative to that of the 185-WT in cortex group. **(D)** The glucose-uptake images in the brain and SUV max detected by PET-CT 2-month post HIE. **(E,H)** TTC staining and quantitative analysis of infarct ratio, respectively, in the rats of 185-WT and 185-KO. Scale bar = 1 cm. Pale color represents infarct area, *n* = 6/group. **(F,I)** HE staining and the cell size analysis of cortex and hippocampus, respectively, in the rats of 185-WT and 185-KO. Scale bar = 50 μm, *n* = 6/group. **(G)** Nissl staining in cortex and hippocampus between 185-WT and 185-KO groups. The white arrow represents surviving neurons. The red arrow represents dark neurons. Scale bar = 100 μm. **(J,K)** Quantitative histogram for total neuron and dark neuron in cortex and hippo in these groups, *n* = 6/group. **(L,M)** The time spent rearing and grooming in the open field test 1 month after HIE, respectively, *n* = 6/group. **(N)** NSS score at 1 month after HIE. **(O)** The duration of on the rotarod bar 1 month after HIE, *n* = 6/group. **(P)** The latency to target for the first 5 days training in the MWM test, *n* = 6/group. **(Q)** Target crossings in MWM test in the 6th day of testing. **(R)** The time spent in the initial, wrong and food arms of Y-maze at 1-month post HIE, *n* = 6/group. HET, heterozygote; 185-WT, miR-185-5p wild type; 185-KO, miR-185-5p knockout; PET-CT, positron emission tomography-computed tomography; SUV, standardized uptake value; HE, hematoxylin-eosin; Hippo, hippocampus. All data are presented as mean ± SD, *p < 0.05.

In the original article, there was a mistake in the legend for Figure 5 as published. The legend of original Figure 5A has been updated owing to the correction made in Figure 5A (details stated in the above section). The correct legend for Figure 5A appears below.

The authors apologize for this error and state that this does not change the scientific conclusions of the article in any way. The original article has been updated.

