# Supplementary material for: Vi4-miR-185-5p-Igfbp3 Network Protects the Brain From Neonatal Hypoxic Ischemic Injury via Promoting Neuron Survival and Suppressing the Cell Apoptosis
Source: Front Cell Dev Biol. 2020 Nov 9;8:529544. doi: 10.3389/fcell.2020.529544 (PMC7688014; doi:10.3389/fcell.2020.529544)
Supplement: Supplementary file 1 [file Image-1.pdf]

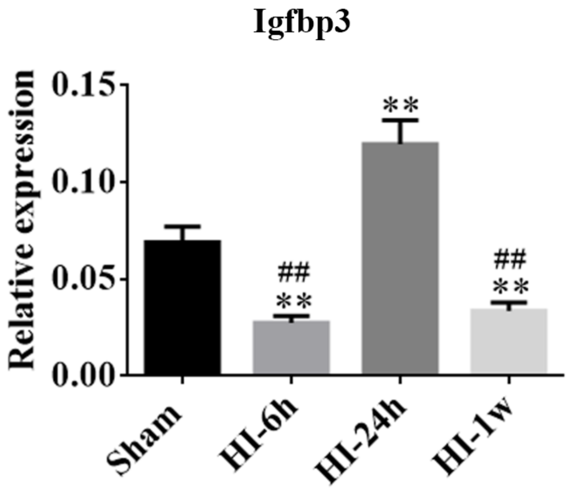

Supplementary Figure1. The expression of Igfbp3 in sham and HI groups. Igfbp3 expression in brain tissues was further validated at 6h, 24h, and 1w after HI, ***P*<0.01 compared with sham group, ##*P*<0.01 compared with HI-24h (24h after HI) group. All data were presented as mean ± SD.
